# Citric acid coated magnetite nanoparticles to overcome intrinsic rifampicin resistance in *Mycobacterium smegmatis*

**DOI:** 10.1186/1471-2334-14-S3-E42

**Published:** 2014-05-27

**Authors:** Priyanka Padwal, Rajdip Bandyopadhyaya, Sarika Mehra

**Affiliations:** 1 Department of Chemical Engineering, Indian Institute of Technology Bombay, Mumbai, India

## Background

The major problem faced in current tuberculosis (TB) therapy is intrinsic drug resistance of mycobacterium. Hence, there is a need to develop ways to overcome these resistance mechanisms. In the current work, we have investigated the effect of citric acid coated magnetite nanoparticles in combination with rifampicin against a wild type strain of *Mycobacterium smegmatis*.

## Methods

We have studied the effect of rifampicin on growth of cells, with and without the nanoparticles. Further, cellular uptake of nanoparticles was studied by transmission electron microscopy. Since, permeability barrier and drug efflux are responsible for intrinsic drug resistance, we have performed accumulation and efflux studies on common efflux pump substrate ethidium bromide (EtBr) by semi automated fluorometric method in the presence and absence of nanoparticles.

## Results

Citric acid coated magnetite nanoparticles exhibited a significant growth inhibition when used in combination with rifampicin. However, nanoparticles alone did not have any effect on mycobacterial growth. Enhanced growth inhibition was seen in the presence of a combination of rifampicin and nanoparticles, which was even more than rifampicin alone at the same concentration. Uptake studies revealed that nanoparticles were internalized by *M. smegmatis*. Transport studies on EtBr demonstrate that, in presence of nanoparticles, the net EtBr accumulation inside the cells was increased.

## Conclusion

Citric acid coated magnetite nanoparticles enhanced the antibacterial effect of rifampicin when used in combination with the drug. Increased accumulation of EtBr in presence of nanoparticles indicates that, one of the possible reasons for this enhanced effect is enhanced intracellular drug accumulation.

